# Eleventh International Medical Congress

**Published:** 1894-02

**Authors:** A. Jacobi

**Affiliations:** 110 W. 34 Street, New York


### ELEVENTH INTERNATIONAL MEDICAL CONGRESS.

A letter directed to the undersigned by the Secretary-General of the Eleventh International Medical Congress, and dated December 19, 1893, contains the following communications:

"American members will pay on the English, French and Italian railways single fares for double journeys, and will obtain a reduction of twenty per cent. on fares for Italian round-trip tickets.

"The documents required for their identification will be sent to you in January, and Americans intending to visit the Congress will have to apply to you for them.

"Full particulars concerning the journey will accompany the documents.

"Messrs. Thos. Cook and Son, London, Paris, Rome, and Naples, shonld be applied to for accommodation and for tickets for the excursions at Rome, Naples, and to Sicily. Such excursions will be arranged at Rome, under the guidance of Mr. Forbes, member of several scientific societies and correspondent of the *Times*—for Naples, three days, including Vesuvius, Pompeii, Capri, Sorrento, Castellamare, Bajae, etc.—for Sicily, ten days from Naples, including Messina, Taormina, Catania, Girgenti, Siracusa, Palermo, and return to Naples.

"The fares for members of the Congress will be considerably reduced, and comprise hotel accommodations, carriages, guides, boats, etc.—about 70 frcs. each, for the three days, and 285 frcs. for the ten days.

"Full particulars concerning these excursions will be contained in a leaflet to be added to the instructions and documents for the journey."

From former communications the following are herewith quoted: The members' fee is five dollars, that of their wives or adult relations two dollars each. Checks or money orders may be sent to Prof. L. Pagliani, Rome, Italy. Credentials have been promised in the near future. When they arrive (none were received last year), they may be too late for many who have started, or are about to start. The undersigned, who is not informed of the cause of delay, proposes to supply, in as official a form as he thinks he is justified in doing, credentials which are expected to be of some practical value. The North German Lloyd has promised to recognize them. It is suggested, besides, that a passport may increase the traveler's facilities.

Only the North German Lloyd (22 Bowling Green) and the Compagnie Générale Transatlantique (3 Bowling Green) have thought fit to grant reductions to Congressists.

The reductions on Italian railways are available from March 1st to April 30th.

A. JACOBI, M. D.,

110 W. 34 Street, New York.

January 11, 1894.

